# The Beneficial Roles of Seaweed in Atopic Dermatitis

**DOI:** 10.3390/md22120566

**Published:** 2024-12-17

**Authors:** Ah-Reum Kim, Myeong-Jin Kim, Jaeseong Seo, Kyoung Mi Moon, Bonggi Lee

**Affiliations:** 1 Department of Smart Green Technology Engineering, Pukyong National University, Busan 48513, Republic of Korea; kimar0107@pukyong.ac.kr; 2 Korean Medicine (KM)—Application Center, Korea Institute of Oriental Medicine, Daegu 41062, Republic of Korea; audwlsabc@naver.com; 3 Department of Food Science and Nutrition, Pukyong National University, Busan 48513, Republic of Korea; syj5528@pukyong.ac.kr (J.S.); omkksm@nate.com (K.M.M.); 4 Marine Integrated Biomedical Technology Center, The National Key Research Institutes, Pukyong National University, Busan 48513, Republic of Korea

**Keywords:** atopic dermatitis, seaweed, anti-inflammatory, immunomodulation, skin barrier, seaweed-derived therapy

## Abstract

Atopic dermatitis (AD) is a chronic, inflammatory skin condition characterized by severe pruritus and recurrent flare-ups, significantly impacting patients’ quality of life. Current treatments, such as corticosteroids and immunomodulators, often provide symptomatic relief but can lead to adverse effects with prolonged use. Seaweed, a sustainable and nutrient-dense resource, has emerged as a promising alternative due to its rich bioactive compounds—polysaccharides, phlorotannins, polyphenols, and chlorophyll—that offer anti-inflammatory, antioxidant, and immunomodulatory properties. This review explores the therapeutic potential of brown, red, and green algae in alleviating AD symptoms, highlighting the effects of specific species, including *Undaria pinnatifida*, *Laminaria japonica*, *Chlorella vulgaris*, and *Sargassum horneri*. These seaweeds modulate immune responses, reduce epidermal thickness, and restore skin barrier function, presenting a novel, safe, and effective approach to AD management. Further clinical studies are needed to confirm their efficacy and establish dosing strategies, paving the way for seaweed-derived therapies as natural alternatives in AD treatment.

## 1. Introduction

Atopic dermatitis (AD) represents a complex, chronic inflammatory skin disorder that disrupts quality of life with symptoms such as pruritus, flare-ups, and sleep disturbances. This disease primarily occurs in the pediatric population, appearing at a frequency of up to 20%. A total of 50–60% of cases occur within the first year of life and 90% of patients experience this condition by the age of five. However, AD can also manifest in adults, with new cases potentially emerging in adulthood [1,2]. Clinical symptoms vary with age (infancy, childhood, adulthood) and degree of skin dryness and can include various skin rashes such as erythema, papules, and vesicular lesions similar to eczema [3]. The most characteristic symptom of AD is persistent pruritus of the skin which interferes with daily activities and can cause insomnia and sleep disorders that degrade the quality of life [4].

The major factors for the onset of AD include genetic factors, defects in epidermal barrier function, changes in immune responses, and dysbiosis in skin microbiology. From an environmental perspective, there are many synergistic factors, such as increased exposure to airborne or food allergens, pollution, infections, antibiotic use, diet, and cosmetics, that contribute to the complex relationships leading to disease onset [5,6,7]. From an immunological perspective, a characteristic phenomenon of AD is the differentiation of CD4 lymphocytes into the T helper 2 (Th2) lineage. The excessive production of Th2 lymphocytes increases the production of cytokines Interleukin (IL)-4, IL-5, and IL-13. The increased secretion of these inflammatory cytokines damages the epidermal barrier and results in primary defects [5,8,9,10]. Factors influencing epidermal destruction such as damage or infection stimulate keratinocytes to produce pro-inflammatory cytokines like IL-25, IL-33, and thymic stromal lymphopoietin (TSLP), which activate Th2-mediated immune responses [9,11]. TSLP activates immature dendritic cells and enhances the maturation of antigen-presenting cells. Furthermore, it promotes eosinophilia activity and chemotaxis while enhancing the expression of IL-4, IL-5, IL-13, and IL-31. IL-25 induces the chemokines necessary for eosinophilia and the recruitment of Th2 cells like eotaxin, thymus activation regulated chemokine (TARC)/CCL17, and macrophage-derived chemokine (MDC). IL-33 stimulates the generation of Th2 response-related cytokines such as IL-4, IL-5, and 13 through its receptor by activating nuclear factor-kappa B (NF-kB) and mitogen-activated protein kinase (MAPK) [5,9,10]. The activation of these Th2-mediated immune responses activates B cells leading to an increase in immunoglobulin (Ig)E secretion which results in mast cell activation along with eosinophils causing skin pruritus increasing cutaneous inflammatory response sensitivity and exacerbating disease [12,13]. This repetitive cycle leads to chronic AD. AD is rapidly increasing in prevalence, severity, and complexity. As there is no clear treatment yet, the burden of medical costs for patients is intensifying, making the development of effective treatments crucial. Treatment methods for AD can be broadly divided into topical and systemic methods [14].

Mild AD is controlled using topical steroids and local immunomodulators. If there is no improvement in pruritus with the use of moisturizers and topical agents, oral antihistamines can be used. Moderate to severe AD involves a combination of topical treatment and systemic treatment. Systemic treatments typically consider short-term systemic steroids or oral cyclosporine. However, if control cannot be achieved with local ointments and cyclosporine, secondary options such as azathioprine (AZP), methotrexate (MTX), mycophenolate mofetil (MMF), antigen-specific immunotherapy, and phototherapy, biological agents including Dupilumab are selected based on the response to treatment, side effects, the patient’s age or health status, pregnancy status, and preferences [15,16]. The downside is that long-term use of local steroids can lead to side effects such as skin atrophy, telangiectasia, hypopigmentation, cataracts, etc., while biological agents including monoclonal antibodies targeting key inflammatory molecules like ILs have shown excellent efficacy with the potential for low side effects in clinical trials.

However, the high cost and accessibility issues pose significant barriers to widespread adoption, limiting utility across larger populations [14]. Furthermore, establishing a universal treatment approach is difficult due to disease heterogeneity, varied clinical presentations, and varied responses to therapies by patients. Therefore, there is growing interest in exploring the potential of natural production and alternative therapies to develop more affordable and accessible treatment options for the management of AD [17].

Seaweed, or macroalgae, is broadly classified into brown, red, and green algae, and while the exact number of species is not officially documented, it is estimated that there are thousands. These marine macroalgae, which are plant-like organisms, typically grow attached to rocks or other solid surfaces in coastal regions. Since the mid-nineteenth century, they have been categorized based on the color of their thalli: brown algae (class *Phaeophyceae*), red algae (phylum *Rhodophyta*), and green algae (phylum *Chlorophyta*) [18]. However, the differences between these groups extend far beyond color. They vary significantly in a range of ultrastructural and biochemical characteristics, such as their photosynthetic pigments, storage compounds, cell wall composition, and the presence or absence of flagella [18]. Other notable distinctions include the ultrastructure of their mitosis, the nature of the connections between adjacent cells, and the detailed structure of their chloroplasts [18].

Seaweed is a widely available resource in oceans globally, particularly in East Asian countries such as Korea, Japan, and China, where it is also consumed as food [19,20]. In addition to its use as a nutritious food source, seaweed has been shown to possess a wide range of biological properties, including anti-inflammatory, antioxidant, anti-cancer, immunomodulatory, anti-tumor, antiviral, antimicrobial, and anti-diabetic activities, making it a subject of active research for its potential applications in the pharmaceutical industry [21].

Moreover, with seaweed’s ability to capture carbon and produce oxygen, it has become a focus of sustainable practices, with many start-ups establishing seaweed farms for large-scale cultivation [22]. This cultivation not only contributes to environmental benefits but also ensures a steady and reliable supply of raw materials for various industries [22]. Given these developments, seaweed offers a secure and environmentally friendly source of raw materials for therapeutic applications, enhancing its appeal as a reliable resource. With its abundance, environmental advantages, and nutrient-rich profile, seaweed holds great potential as an effective and safe therapeutic agent. Seaweed is particularly rich in bioactive compounds like polysaccharides, polyphenols, phlorotannins, and chlorophyll, and is nutritionally dense, containing proteins, vitamins, and minerals. As a result, research aimed at developing seaweed-based treatments for various diseases is ongoing, with numerous studies already confirming the diverse bioactivities of seaweed extracts, isolated compounds, and their derivatives [23,24].

In this review, we will explore the current research trends on seaweed as a potential treatment for AD and evaluate whether seaweed can be developed into an effective therapeutic agent for managing AD symptoms.

## 2. Potentials of Seaweed in the Treatment of Atopic Dermatitis

### 2.1. Phaeophyceae (Brown Algae)

#### 2.1.1. *Undaria pinnatifida*

*Undaria pinnatifida*, Wakame, is a brown seaweed commonly found in the coastal waters of East Asia, including Japan, Korea, and China, and is widely used as a food source rich in nutrients like vitamins and minerals. Research highlights its antioxidant, anti-inflammatory, and anticancer properties, attributed to compounds such as fucoxanthin and fucoidan [25].

Research exploring fucoidan’s anti-atopic properties employed NC/Nga mice and human keratinocyte models to evaluate its therapeutic potential in treating AD [26]. In the NC/Nga mouse model, 1-Chloro-2,4-dinitrobenzene (DNCB) was applied to the dorsal skin to induce AD-like conditions. From the fifth week, fucoidan was administered orally, with dexamethasone as a comparison [26]. Fucoidan treatment led to marked reductions in dermatitis severity scores versus the untreated AD group, showing observable improvements such as decreased scratching behavior and reduction in visible skin symptoms, indicative of inflammation control. Skin tissue histology further showed reduced epidermal thickness and lower mast cell infiltration in the fucoidan-treated group, mirroring the anti-inflammatory effects seen with dexamethasone [26]. Serum analyses revealed fucoidan significantly lowered histamine and moderately decreased IgE levels, both of which are key markers in assessing AD severity and allergic responses [26]. In complementary tests using TNF (Tumor Necrosis Factor) -α and IFN (interferon)-γ stimulated human keratinocytes, fucoidan treatment resulted in a dose-dependent reduction in the mRNA expression of chemokines TARC/CCL17, MDC, and RANTES. Since these chemokines drive immune cell migration to inflamed areas, this suggests fucoidan’s potential to reduce immune cell recruitment at AD lesion sites [26].

Altogether, these findings highlight fucoidan’s promising capacity to alleviate AD-like symptoms by controlling inflammation, modulating immune response markers, and inhibiting chemokine production linked to AD inflammation.

#### 2.1.2. *Costaria costata*

*Costaria costata* (*C. costata*), a brown alga commonly found along the coastlines of Korea, China, and Japan, has recently been studied for its anti-cancer properties and protective effects against CCL4-induced liver injury [27,28]. One study investigates the therapeutic potential of *C. costata* extracts in improving DNCB-induced AD in a mouse model [29]. The *C. costata* extract was obtained using 10% ethanol (CCE10) and administered through the diet [29]. Forty mice were divided into five groups of eight: a normal Balb/C group, an AD control group induced with 0.1% (*w*/*v*) DNCB, a positive control group treated with 66.6 mg/kg of CJLP133 after DNCB induction, and two experimental groups receiving either 100 mg/kg (CCE10 100) or 300 mg/kg (CCE10 300) of CCE10-supplemented diet [29]. Histopathological analysis of the dorsal skin and ear tissue revealed that dermal thickening, increased dead skin cell layers, and inflammatory cell infiltration in the AD control group were alleviated in both the CCE10 100 and 300 groups [29]. The treatment also influenced several immune responses. Eotaxin (a CC chemokine subfamily of eosinophil chemotactic proteins) and TARC mRNA levels, which were significantly elevated in the AD control group, were notably reduced in the CCE10-treated groups, showing results similar to the positive control group [29]. Additionally, splenic T cell proliferation decreased, and B cell proliferation increased in the AD control group. However, in the CCE10-treated groups, T cell proliferation increased while B cell proliferation decreased [29]. Furthermore, cytokine analysis demonstrated a shift in immune balance. T helper 1 (Th1) cytokines (IL-2, IFN-γ) were reduced, while Th2 cytokines (IL-4, IL-10) and inflammatory cytokines (TNF-α, IL-6) were elevated in the AD control group [29]. CCE10 treatment reversed these changes, promoting Th1 cytokine production and suppressing Th2 and inflammatory cytokines [29]. Serum IgE and histamine levels, which were significantly elevated in the AD control group, were also reduced with CCE10 treatment, along with decreased mast cell infiltration [29].

These findings suggest that *C. costata* helps regulate immune responses in AD by reducing chemokine expression, normalizing T and B cell proliferation, balancing cytokine production, and lowering IgE, histamine levels, and mast cell infiltration.

#### 2.1.3. *Laminaria (Saccharina) japonica*

*Laminaria japonica* (*L. japonica*), a type of brown algae referred to as “Gonpo” or “Dasima” in Korea, “Kunbu” or “Haidai” in Chinese, and “Kelp” in Europe and North America [30], has been traditionally used in oriental medicine to address a variety of ailments. [31,32]. Previous studies have demonstrated that *L. japonica* possesses various biological activities, such as anti-tumor, anti-coagulant, and anti-viral properties, along with the ability to induce hypoglycemia and hypolipidemia, with confirmed immunomodulatory properties in both in vitro and in vivo research [31,33,34,35]. One study examined the therapeutic potential of *L. japonica* water extract (LJWE) on DNCB-sensitized NC/Nga mice and human keratinocytes [36]. The study demonstrated that LJWE can reduce inflammatory chemokines, including RANTES, TARC, and IL-8, in TNF-α/IFN-γ-stimulated human keratinocytes by inactivating the signal transducer and activator of transcription (STAT) 1 through the inhibition of p38 MAPK phosphorylation. This led to notable improvements in skin hydration, decreased dermatitis severity, and reduced inflammatory mediator levels in DNCB-sensitized NC/Nga mice. [36]. Another study investigated the effects of *L. japonica* extract on AD using both in vivo and in vitro models. Mice treated with the extract (10 or 100 mg/kg) for two weeks exhibited significant improvements in DNCB-induced AD symptoms, such as excoriation, erythema, and skin thickening [37]. The extract also reduced serum histamine and IgE levels at the higher dosage and decreased IL-6 and IL-8 production by inhibiting the nuclear translocation of NF-κB p65 in TNF-α/IFN-γ-stimulated cells (at concentrations of 1 mg/mL) [37].

Overall, *L. japonica* extract shows promise for treating AD by alleviating clinical symptoms, lowering serum histamine and IgE levels, and reducing inflammatory cytokine production through modulation of the NF-κB and STAT1 pathways.

#### 2.1.4. *Ecklonia cava*

*Ecklonia cava* (*E. cava*), a type of brown algae from the class *Phaeophyceae* and family *Laminariaceae*, flourishes in the rocky coastal regions of Korea and Japan [38,39]. This perennial seaweed is nutrient-dense and contains a variety of phytochemicals, including phlorotannins, sulfated polysaccharides (such as fucoidans), peptides, and carotenoids [40]. Phlorotannins, polymers of the benzenetriol compound phloroglucinol, exhibit different degrees of polymerization and bonding. Specific phlorotannins present in *E. cava* include dieckol, phlorofucofuroeckol, 6,6′-bieckol, eckol, eckstolonol, phlorofucofuroeckol A, 7-phloroeckol, 6,6′-bieckol, 8,8′-bieckol, and triphlorethol A [40]. These compounds or extracts from *E. cava* have shown antioxidant [41], anti-inflammatory [42], anti-allergic [43], anti-diabetic [41], anti-cancer [44], hepatoprotective [45], antihypertensive [46], and neuroprotective properties [47].

Various studies showed the anti-atopic effects of *E. cava*-derived compounds. Dieckol isolated from *E. cava* reduced MDC/CCL22 production in IFN-γ (10 ng/mL)-stimulated HaCaT human keratinocyte cells in a dose-dependent manner [38]. Specifically, dieckol (at 5 and 10 μM) inhibited the phosphorylation and nuclear translocation of STAT1 [38]. Other in vitro studies also showed the beneficial effects of *E. cava*-derived compounds. Eckol treatment (at 25, 50, 100 μg/mL) suppressed the mRNA expression of TNF-α/IFN-γ-induced pro-inflammatory cytokines (IL-1β, IL-4, IL-5, IL-6, IL-13, IFN-γ, and TNF-α) and chemokines (IL-25, IL-33, and TSLP) in HaCaT cells [39]. TNF-α/IFN-γ treatment resulted in the phosphorylation of p38 and the extracellular signal-regulated kinase (ERK), which was significantly inhibited by eckol in a dose-dependent manner [39]. These results indicate that eckol suppresses TNF-α/IFN-γ-induced MAPK activation in HaCaT cells. Additionally, eckol inhibited the nuclear translocation of NF-κB p65 in TNF-α/IFN-γ-stimulated HaCaT cells, as shown by western blot analysis [39]. These findings indicate that eckol inhibits TNF-α/IFN-γ-induced phosphorylation and nuclear translocation of NF-κB in HaCaT cells [39]. In another study, TNF-α/IFN-γ-induced phosphorylation of NF-κB in HaCaT cells was inhibited by eckol (at 100 μg/mL) [48]. Additionally, eckol not only reduces the phosphorylation of Janus kinase 1 (JAK1) increased by TNF-α/IFN-γ, but also inhibits the activity of its downstream regulator, STAT, at the same concentration [48]. JAK1 is activated by the binding of IFN-γ to its receptor, which allows it to phosphorylate tyrosine residues on the cytokine receptor and other JAKs, creating docking sites [49]. This process, in turn, recruits and phosphorylates STAT1. Once activated by JAK, STAT1 forms dimers with another STAT1 and translocates to the nucleus, where it binds to DNA to regulate the transcription of genes involved in the production of cytokines and chemokines.

To sum up, Dieckol from *E. cava* reduces MDC/CCL22 production and inhibits STAT1 activation in IFN-γ-stimulated HaCaT cells, exerting anti-inflammatory effects. Similarly, eckol suppresses pro-inflammatory cytokine and chemokine expression by inhibiting the MAPK and NF-κB pathways, as well as JAK1/STAT1 signaling, in TNF-α/IFN-γ-stimulated HaCaT cells. Based on these results, it is considered that *E. cava* has potential as a material for improving AD.

#### 2.1.5. *Cladosiphon okamuranus*

*Cladosiphon okamuranus* (*C. okamuranus*), commonly known as *Okinawa Mozuku*, is a brown seaweed endemic to the Okinawa region in Japan, where it is cultivated for its nutritional and economic value [50]. This seaweed has earned recognition in Japanese cuisine due to its high content of bioactive compounds, notably fucoidan—a sulfated polysaccharide known for its diverse health-promoting effects [50]. Research has linked the fucoidan in *C. okamuranus* to multiple physiological benefits, including antioxidant, anti-inflammatory, and immune-regulating activities [51].

In this study, *C. okamuranus* fucoidan was evaluated for its efficacy in mitigating AD symptoms in a DNCB-induced AD model using BALB/c mice [52]. The mice were treated with the fucoidan at doses of 200, 400, and 800 mg/kg, administered orally, alongside a control group and a dexamethasone-treated group for comparison. Results showed that fucoidan treatment significantly reduced AD-related clinical symptoms, as evidenced by lowered SCORAD (scoring atopic dermatitis) scores and a decrease in dorsal skin thickness, cell infiltration, and other histopathological markers of AD [52]. Fucoidan’s therapeutic effects inhibited mast cell degranulation and histamine release, which are key players in the allergic response characteristic of AD [52]. This was observed in cell-based assays using P815 mast cells, where fucoidan reduced IL-4 and histamine release in a dose-dependent manner [52]. Fucoidan also suppressed key Th2 cytokines, IL-4 and IL-13, which are associated with the allergic inflammatory response in AD [52]. The levels of pro-inflammatory cytokines, such as IL-6 and IL-1β, were also reduced, suggesting that fucoidan can help moderate both allergic and inflammatory pathways [52]. Further immunological analysis showed that fucoidan-treated mice exhibited lower IgE levels in serum, a critical indicator of AD severity, and reduced eosinophil infiltration in affected skin tissues [52]. Additionally, fucoidan administration reversed the Th2 polarization observed in AD by restoring the balance between Th1 and Th2 responses, indicated by normalized levels of Th1 cytokines (e.g., IFN-γ) and reduced Th2-dominant cytokines [52]. Fucoidan’s influence on skin homeostasis was also notable, as it lowered the levels of TSLP and IL-22, cytokines that contribute to epidermal hyperplasia and skin barrier dysfunction in AD [52].

In another investigation, *C. okamuranus* fucoidan, sourced from Kanwa Healthcare Ltd. (New Taipei, Taiwan), demonstrated that it significantly inhibited nitric oxide (NO) production in LPS-induced RAW264.7 cells, with a dose-dependent reduction observed at concentrations as low as 31.25 μg/mL [53]. In a DNCB-induced mouse model, topical application of fucoidan creams (3.5% and 7%) substantially improved AD symptoms, as evidenced by lower SCORAD scores and attenuated lymph node proliferation, suggesting reduced allergic responses [53]. Additionally, fucoidan treatment reduced serum IgE levels, epidermal thickness, eosinophil infiltration, and inflammatory responses [53]. Systemic immunity analysis showed that fucoidan downregulated the expression of T-cell co-stimulatory molecules CD80 and CD86 while upregulating inhibitory receptors CD273 and CD274 [53]. Fucoidan also modulated AD-associated cytokines (IL-4, IL-5, IL-22, IL-33, and TSLP), promoting an anti-inflammatory profile by increasing transforming growth factor (TGF) -β1 levels [53]. Moreover, fucoidan reduced Th22 cell and memory B cell populations, highlighting its immunomodulatory effects [53].

In conclusion, the findings from both studies underscore the significant potential of fucoidan from *C. okamuranus* in addressing the symptoms of AD through their demonstrated abilities to modulate immune responses and cytokine profiles. These compounds offer promising avenues for the management and treatment of this chronic skin condition.

#### 2.1.6. *Fucus vesiculosus*

*Fucus vesiculosus* (*F. vesiculosus*), commonly referred to as bladder wrack, is a brown macroalga primarily distributed in the Northern Hemisphere, flourishing along intertidal zones in the Baltic and North Seas, and along the Atlantic coastlines of Europe and North America [54,55]. This species is nutritionally notable for its high content of dietary fiber, essential minerals, and vitamins, while being naturally low in fat, making it an excellent nutritional resource [54,55]. Traditional uses of Fucus species, especially in regions of East Asia and Western Europe, reflect its therapeutic potential; it has been widely used in folk medicine, particularly valued for its iodine content to treat goiter and thyroid-related issues [54,55]. *F. vesiculosus* also boasts a potent profile of bioactive compounds like phlorotannins, fucoidans, and fucoxanthin, which contribute to its antioxidant, anti-inflammatory, antimicrobial, and anticancer properties [54,55].

A study investigated the therapeutic potential of *F. vesiculosus* fucoidan, a sulfated polysaccharide, in alleviating AD-like symptoms induced by dinitrofluorobenzene (DNFB) in Balb/c mice [56]. Fucoidan, known for its biological activities, acts as a natural ligand for class A scavenger receptors (SR-A), crucial in immune regulation [57,58,59,60]. In DNFB-induced AD mice, fucoidan treatment (50 μL of 0.2% fucoidan) reversed the reduction in SR-A mRNA levels and improved symptoms such as erythema, edema, and dryness, while reducing epidermal thickness, inflammatory cell infiltration, and serum IgE and IL-4 levels, indicating a reduction in allergic activity [56]. At the molecular level, fucoidan decreased pro-inflammatory cytokines like TNF-α and IL-12 and increased anti-inflammatory cytokines IL-10 and TGF-β, shifting the immune response toward an anti-inflammatory profile [56]. It also reduced CD4+ T cell infiltration into the ear tissues, with a negative correlation between SR-A expression and CD4+ T cell presence, indicating fucoidan’s potential role in suppressing CD4+ T cell proliferation via SR-A interaction [56]. The study further showed that fucoidan reduced inflammatory Th1 and Th17 cell populations while boosting regulatory T cells (Tregs), highlighting its role in controlling immune responses [56]. Fucoidan also reduced CD4+ T cell infiltration in the skin lesions and spleens of AD mice [56].

In summary, the study demonstrated that fucoidan from *F. vesiculosus* alleviated AD symptoms by modulating immune responses, increasing SR-A expression, reducing inflammatory cytokines, and promoting an anti-inflammatory profile, especially through the enhancement of Treg populations. These findings highlight fucoidan’s potential as a therapeutic agent for AD through immune system modulation.

#### 2.1.7. *Sargassum*

*Sargassum* is a genus of brown seaweed rich in bioactive compounds such as fucoidan, polyphenols, and polysaccharides, which are well known for their anti-inflammatory, antioxidant, and immune-modulating properties. These compounds are particularly promising for the treatment of AD.

The ethanol extract of *Sargassum horneri* (*S. horneri*) significantly reduced the levels of pro-inflammatory cytokines, including IL-25, IL-33, thymic stromal TSLP, IL-1β, IL-4, IL-6, IL-13, IFN-γ, and TNF-α in TNF-α/IFN-γ-induced HaCaT keratinocyte models [61]. Furthermore, *S. horneri* ethanol extract (EE) inhibited the expression of inflammatory chemokines, such as eotaxin, MDC, and TARK, contributing to the reduction of inflammation at the cellular level. *S. horneri* EE also reduced the production of inflammatory mediators through the inhibition of the NF-κB pathway and suppressed the activation of the ERK/p38 signaling pathway, thereby enhancing the antioxidant defense system mediated by nuclear factor erythroid-2-related factor (Nrf2)/heme oxygenase-1 (HO-1). In a 12-O-tetradecanoylphorbol-13-acetate (TPA) -induced ear edema mouse model, *S. horneri* EE reduced ear thickness, inflammatory cell infiltration, hyperplasia, and hyperkeratosis, demonstrating its anti-inflammatory effects. These findings suggest that *S. horneri* EE has anti-inflammatory potential in both TNF-α/IFN-γ-induced keratinocyte inflammation and TPA-induced ear edema models [61].

Another study evaluated the effects of *S. horneri* EE on NC/Nga mice with AD induced by DNCB [62]. *S. horneri* EE treatment reduced epidermal thickness and hyperplasia, which were exacerbated by DNCB exposure. *S. horneri* EE significantly inhibited mast cell proliferation and decreased the serum levels of IgG₁ and IgG₂_a_ as well as the expression of IL-13 in CD4⁺ cells, which are key elements involved in DNCB-induced AD. *S. horneri* EE also inhibited the expression of TSLP produced by keratinocytes, preventing skin barrier damage. Additionally, reduced levels of filaggrin, a crucial protein for maintaining proper skin barrier function, were significantly improved by *S. horneri* EE treatment in DNCB-induced AD mice. These results indicate that *S. horneri* EE protects NC/Nga mice from DNCB-induced AD by promoting skin barrier function, regulating inflammatory cytokines, and modulating immune responses [62]. The oral administration of *S. horneri* also showed promising effects in improving AD induced by house dust mite (HDM)/DNCB in NC/Nga mice. The results showed that oral *S. horneri* administration significantly reduced dermatitis symptoms and inflammatory cytokine production [63]. The extract inhibited the production of Th2 cytokines (IL-4, IL-5, IL-6, IL-10, IL-13 and IFN-γ) levels. Additionally, it reduced inflammatory cell infiltration and epidermal thickness, leading to overall improvement in skin health. The decrease in serum IgE levels further indicated the suppression of allergic responses, supporting the role of orally administered *S. horneri* as an effective natural therapy for AD [63].

Additionally, in a DNCB-induced BALB/c mouse model, *S. horneri* hot water extract (HWE) exhibited anti-inflammatory and immune-modulating effects [64]. *S. horneri* HWE was shown to inhibit Th2-related cytokines (IL-4, IL-5) in a dose-dependent manner and increase Th1 cytokine (IFN-γ) production, restoring the Th1/Th2 balance [64]. Furthermore, *S. horneri* HWE significantly decreased serum IgE levels, thereby alleviating allergic reactions related to AD [64]. Histological analysis revealed that *S. horneri* HWE reduced epidermal thickness and inflammatory cell infiltration, contributing to improved skin barrier function and AD symptom reduction [64].

These findings suggest that *S. horneri* HWE has potential as a natural treatment for inflammatory skin conditions such as AD, indicating that both *S. horneri* EE and HWE exert anti-inflammatory effects through multiple mechanisms [61,62,63,64]. TNF-α and IFN-γ are key inflammatory cytokines that promote skin inflammation and activate immune responses, but *S. horneri* extracts have been shown to block these cytokine signaling pathways and inhibit the NF-κB pathway, thereby reducing the production of inflammatory mediators. Moreover, by increasing the expression of HO-1, *S. horneri* extracts enhance anti-inflammatory effects through multiple pathways.

*S. horneri* is not the only type of *Sargassum* showing inhibitory effects on AD symptoms. Recent studies have found that *Sargassum fulvellum* (*S. fulvellum*) EE and its active component, grasshopper ketone, exhibit anti-atopic effects. This study demonstrated that *S. fulvellum* EE effectively alleviated symptoms of AD in both in vivo and in vitro models [65]. Treatment with the extract significantly improved major AD symptoms, such as erythema, edema, and skin dryness, in DNCB-induced BALB/c mice models and reduced inflammatory cell infiltration and epidermal thickness. Additionally, *S. fulvellum* EE significantly suppressed the expression of pro-inflammatory cytokines such as IL-4, IL-5, and IL-13, which play a role in the pathogenesis of AD. In particular, grasshopper ketone contributed to the regulation of immune responses and reduction in serum IgE levels, helping to alleviate allergic inflammation [65].

Moreover, a compound isolated from *Sargassum muticum* (*S. muticum*), Apo-9′-fucoxanthinone, also demonstrated anti-inflammatory effects against AD [66]. The topical application of Apo-9′-fucoxanthinone reduced the symptoms of erythema, edema, and skin thickness, while histological analysis showed decreased inflammatory cell infiltration in the skin tissue [66]. This compound significantly inhibited the expression of inflammatory cytokines, including TNF-α, IFN-γ, and IL-4, and reduced serum IgE levels, indicating suppression of allergic responses [66]. Additionally, Apo-9′-fucoxanthinone positively affected skin barrier function and regulated immune cell activation, suggesting its potential for the long-term suppression of inflammatory responses [66].

Furthermore, *Sargassum hemiphyllum* (*S. hemiphyllum*) has been traditionally used in Korean medicine to treat allergic diseases [67]. Recent studies have investigated the effects of *S. hemiphyllum* methanol extracts (ME) on allergic responses in AD [67]. This extract inhibited histamine and β-hexosaminidase release induced by compound 48/80 in rat peritoneal mast cells and also reduced the expression of IL-8 and TNF-α in HMC-1 cells induced by phorbol 12-myristate 13-acetate (PMA) and A23187 [67]. Additionally, *S. hemiphyllum* ME inhibited passive cutaneous anaphylaxis induced by anti-dinitrophenyl IgE antibodies and blocked the increase in NF-κB protein levels induced by TNF-α, thereby demonstrating its effectiveness in interrupting inflammatory signaling pathways [67]. These findings suggest that *S. hemiphyllum* ME reduces allergic inflammatory responses by suppressing mast cell activation and the production of inflammatory mediators [67].

The ethanolic extract of *Sargassum serratifolium* (*S. serratifolium*), which is known for its diverse bioactive components, has demonstrated promising therapeutic effects in a mouse model of AD induced by DNCB [68]. This was further supported by in vitro studies involving RAW 264.7 macrophages and HaCaT cells. *S. serratifolium* EE treatment improved AD symptoms in both BALB/c and HR-1 mice, including improvements in histological features such as reduced epidermal thickness and decreased mast cell infiltration—key indicators of inflammation in AD. In addition to improving clinical symptoms, *S. serratifolium* EE also demonstrated a trend toward lowering serum IgE levels and modulating inflammation-related cytokine changes, which are closely associated with allergic responses in AD. Furthermore, in RNA sequencing analyses of LPS-stimulated macrophages treated with *S. serratifolium* EE, it was found that *S. serratifolium* EE has the capability to inhibit a wide range of inflammation-related signaling pathways. The study highlighted improvements in the mRNA expression of genes such as Chi3l3, Ccr1, and Fcεr1a in the skin, all of which play roles in immune regulation and inflammation. The compounds from *S. serratifolium* EE, including sargachromanol (SCM), sargaquinoic acid (SQA), and sargahydroquinoic acid (SHQA), were shown to reduce inflammatory responses in LPS-treated RAW264.7 macrophages, further demonstrating the anti-inflammatory effects of *S. serratifolium* EE. [68].

In conclusion, studies on *Sargassum* species highlight their strong anti-inflammatory and skin barrier-restoring effects, suggesting they hold great potential as a therapeutic option for AD. By targeting multiple inflammatory pathways, *Sargassum* offers a promising, safe, and sustainable alternative for managing chronic conditions like AD, especially given the limitations of conventional therapies.

#### 2.1.8. *Hizikia (Sargassum) fusiformis*

*Hizikia fusiformis Okamura* (*H. fusiformis*), also known as *Sargassum fusiforme* (Harvey) Setchell, is a prevalent brown seaweed found along the rocky coastlines of Korea, Japan, and China. Traditionally consumed in Asia for its nutritional benefits, *H. fusiformis* has attracted considerable attention owing to its promising prospects in the pharmaceutical and industrial sectors. Extensive research has highlighted the diverse pharmacological activities of *H. fusiformis* extracts, including anti-leukemia, anti-tumor, anti-fatigue, anti-diabetic, anti-aging, immunomodulatory, anti-inflammatory, and antioxidant properties [69,70,71,72,73,74,75,76].

A comprehensive study demonstrated that a butanoic acid-rich fraction, known for its anti-inflammatory and anti-atopic properties [77], was isolated from the ethyl acetate (EA) extracts of *H. fusiformis* [69]. These fractions effectively inhibited T cell activation by preventing the dephosphorylation of nuclear factor of activated T cell (NFAT), as evidenced by electrophoretic mobility shift assays [69]. Consequently, helper T cell-dependent cytokines such as IL-2, IL-4, and IFN-γ were significantly suppressed when activated with an anti-CD3 antibody [69]. Histological analysis of skin from DNCB-treated mice revealed increased thickness and cell infiltration, both of which were significantly reduced by treatment with the EA extract at doses of 1.25 and 2.5 mg/kg [69]. Additionally, *H. fusiformis* EA extract markedly inhibited IL-4 and IL-5 mRNA expression in splenocytes, indicating a strong suppression of T cell activation [69].

Fucosterol, another compound purified from *H. fusiformis*, reduced the production of NO induced by LPS in RAW 264.7 macrophage cells [78]. In a DNCB-induced AD mouse model, oral administration of fucosterol alleviated key AD symptoms, including erythema, scratching, and inflammatory cell infiltration. Histological analysis showed that fucosterol decreased epidermal thickness and reduced the presence of mast cells, comparable to the effects of dexamethasone, a standard anti-inflammatory drug [78]. Furthermore, fucosterol demonstrated immunomodulatory effects by suppressing IL-4 and TNF-α levels, key markers of Th2 and inflammatory responses, respectively, while enhancing IFN-γ production, a Th1 cytokine. This shift in cytokine balance, achieved in splenocyte cultures with a reduction in IL-4 and TNF-α compared to the concanavalin A control, contributed to the lowering of serum IgE, an indicator of allergic response severity. Together, these effects indicate that *H. fusiformis* fucosterol may help rebalance the Th1/Th2 immune response, providing both anti-inflammatory and anti-allergic benefits relevant to AD pathogenesis [78].

Put together, *H. fusiformis* offers a promising source of bioactive compounds, particularly butanoic acid and fucosterol, with significant potential for anti-inflammatory and anti-allergic therapies in chronic skin conditions like AD due to their immunomodulatory and anti-inflammatory effects.

#### 2.1.9. *Ishige okamurae Yendo*

*Ishige okamurae* (*I. okamurae*), belonging to the *Fucaceae* family, is brown algae commonly found along the southern coastlines, particularly abundant in tranquil intertidal zones, with significant culinary importance in Korea as one of the most consumed edible seaweeds. Numerous bioactive compounds, such as polysaccharides, pigments, and phenolic compounds, have been identified from this seaweed species [79,80,81,82,83]. A study explores the anti-inflammatory and anti-atopic effects of *I. okamurae* EE and its active phenolic compound, diphlorethohydroxycarmalol (DPHC), in cellular and animal models of AD. Notably, DPHC, isolated from *I. okamurae*, has attracted interest for its broad therapeutic benefits, including skin protection and anti-inflammatory, anti-diabetic, antioxidant, and anti-melanogenic properties. [84,85,86,87,88].

Under TNF-α/IFN-γ-induced inflammatory conditions, pretreatment with *I. okamurae* EE resulted in a dose-dependent reduction in pro-inflammatory cytokines and chemokines such as IL-1β, IL-6, IL-8, TNF-α, TARC/CCL17, and CCL22/MDC. *I. okamurae* EE also significantly inhibited the phosphorylation of essential inflammatory signaling molecules, including MAPKs, STAT-1, and NF-κB—key pathways implicated in AD pathogenesis—suggesting that the extract reduces inflammation through targeted modulation of these signaling processes [83]. In a DNCB/HDM-induced AD mouse model, *I. okamurae* EE improved skin lesions, reduced ear edema, and decreased immune cell and mast cell infiltration. Additionally, the extract lowered serum levels of IgE, IgG1, and IgG2a, indicators of Th2 and Th1 immune responses. Furthermore, it downregulated mRNA levels of pro-inflammatory cytokines and chemokines in ear tissues, reinforcing its anti-inflammatory potential in alleviating AD lesions. [83]. DPHC exhibited similar anti-inflammatory properties. It effectively inhibited the same signaling pathways (MAPKs, STAT-1, NF-κB) and suppressed pro-inflammatory gene expression in TNF-α/IFN-γ-stimulated HaCaT keratinocyte cells. In the AD mouse model, DPHC also reduced serum levels of IgE, IgG1, and IgG2a and downregulated cytokine and chemokine expression in ear tissues, highlighting its capacity to modulate Th2-driven responses in AD. [83]. Overall, this study supports EE and DPHC from *I. okamurae* as promising candidates for AD therapy, demonstrating their ability to reduce pro-inflammatory cytokine levels, regulate immune responses, and mitigate AD symptoms through the inhibition of inflammatory signaling pathways.

Table 1 offers a streamlined summary of the main findings from the studies discussed, providing a quick reference to the core results highlighted in this section (Table 1).

### 2.2. Rhodophyta (Red Algae)

#### 2.2.1. *Sarcodia suiae* sp.

*Sarcodia suiae* sp. (*S. suiae*), a species of red macroalgae belonging to the phylum *Rhodophyceae*, is primarily found along the coast of Taiwan. Known for its high micronutrient content, it is commonly used as food or as a food additive [89,90]. In addition, the water extract of *S. suiae* acts as an immunostimulant, activating Th1-like immune responses [91].

This study evaluated the effects of the ethyl acetate fraction (EAF) of *S. suiae* on DNCB-induced AD-like lesions [90]. In mice with DNCB-induced AD, the clinical dermatitis scores increased compared to the control group. However, treatment with EAFL (*n* = 6, DNCB + 50 µg EAF in 100 µL vehicle) and EAFH (*n* = 6, DNCB + 200 µg EAF in 100 µL vehicle) alleviated dryness, erythema, edema, and hyperkeratosis, resulting in lower clinical dermatitis scores compared with the vehicle group (1% methylcellulose). Furthermore, *S. suiae* EAF significantly suppressed the elevated serum IgE levels when compared to the control group. Additionally, *S. suiae* EAF reduced the size and weight of the spleen and inguinal lymph nodes in DNCB-induced AD mice. Histopathological observations revealed that the *S. suiae* EAF significantly and dose-dependently decreased mast cell infiltration. Immunofluorescence staining also confirmed that PD1 effectively improved the deficiencies in claudin-1 and filaggrin. These findings suggest that the bioactive compounds in PD1, including its immunomodulatory properties, may contribute to the suppression of inflammatory responses and play a crucial role in alleviating AD symptoms in DNCB-induced mice.

#### 2.2.2. *Chondria crassicaulis*

*Chondria crassicaulis* (*C. crassicaulis*) is a red alga belonging to the *Rhodomelaceae* family, found in the coastal waters of Korea and China. It typically grows attached to rocks or other seaweeds along the shore, exhibiting a reddish-brown or purple color. In addition, *C. crassicaulis* extract has been shown to inhibit the production of pro-inflammatory cytokines TNF-α, IL-1β, and IL-6 in LPS-stimulated macrophages.

This study investigated the effect of EE from *C. crassicaulis* on degranulation in AD-like skin lesions induced by DNCB in mice [92]. The composition of the *C. crassicaulis* EE was determined to be 7.59% moisture, 46.16% ash, 19.41% crude protein, 0.82% crude fat, and 26.29 mg glucose corresponding to total sugar. Additionally, *C. crassicaulis* EE contained 1.20 mg gallic acid (total polyphenol content) and 0.59 mg quercetin (total flavonoid content), confirming its potential for strong antioxidant properties, similar to other seaweeds [92]. In an animal model, the effects of *C. crassicaulis* EE on AD were evaluated by inducing AD using DNCB in BALB/c mice [92]. The application of *C. crassicaulis* EE at a concentration of 30 mg/mL for two weeks significantly improved AD symptoms, markedly reducing dermatitis severity, including erythema, scarring, swelling, and scratching [92]. Furthermore, *C. crassicaulis* EE treatment significantly lowered total IgE levels in the serum, suggesting that it alleviates the physical symptoms of AD and potentially mitigates the overall allergic response [92]. In vitro studies using RBL-2H3 cells demonstrated that *C. crassicaulis* EE treatment suppressed the release of β-hexosaminidase from mast cells, an indicator of allergic reactions [92]. This indicates its potential to reduce allergic inflammation. Additionally, it altered the cytokine secretion profile of spleen cells. Specifically, it reduced levels of Th2 cytokines, such as IL-4 and IL-5, which are typically elevated in AD, while increasing levels of Th1 cytokines like IFN-γ [92]. These changes are important for alleviating the inflammatory processes underlying AD. These results suggest that *C. crassicaulis* EE treatment may effectively alleviate the symptoms of AD by reducing inflammatory responses.

#### 2.2.3. *Porphyra* (*Pyropia*) *yezoensis*

*Porphyra* is a red alga belonging to the *Porphyraceae* family, commonly found along the coasts of Korea, Japan, and China. It is widely consumed throughout Asia as a source of processed food and health-promoting compounds, providing significant nutritional and economic value [93]. *Porphyra* contains a diverse range of bioactive compounds, including proteins, minerals, dietary fiber, polyunsaturated fatty acids, carotenoids, sugars, and mycosporine-like amino acids [94]. It is widely consumed for its numerous health benefits, such as its antioxidant, anti-inflammatory, anticancer, antihypertensive, UV-protective, and tissue-healing effects [95,96,97,98,99,100].

A study investigated the anti-AD effects of *Porphyra yezoensis* (*P. yezoensis*) extract on mRNA and protein levels of inflammatory chemokines, particularly TARC/CCL17 and MDC [93]. The extract significantly inhibited the mRNA expression of TARC and MDC in a dose-dependent manner in IFN-γ-stimulated HaCaT cells. Additionally, *P. yezoensis* extract inhibited TARC mRNA expression in TNF-α-stimulated HaCaT cells, though its inhibitory effect on MDC was weaker. Beyond mRNA expression, *P. yezoensis* extract treatment significantly reduced the secretion of TARC and MDC into the culture medium compared to IFN-γ or TNF-α stimulation alone. These results suggest that *P. yezoensis* extract may suppress the release of inflammatory chemokines by regulating mRNA expression [93]. Further analysis through Western blotting revealed that *P. yezoensis* extract inhibited the phosphorylation of MAPK (ERK, JNK, and p38) in IFN-γ or TNF-α-stimulated HaCaT cells [93]. This inhibition suggests that *P. yezoensis* extract exerts its anti-inflammatory effects, at least in part, by regulating the MAPK signaling pathway [93]. In addition, *P. yezoensis* extract inhibited IFN-γ and TNF-α-induced NF-κB activation in HaCaT cells [93]. This inhibition was associated with a reduction in NF-κB/p65 phosphorylation and preservation of IκB-α levels, indicating that *P. yezoensis* extract attenuates inflammatory responses by interfering with both the MAPK and NF-κB signaling pathways [93]. HPLC analysis of the carotenoid composition of *P. yezoensis* extract identified significant levels of astaxanthin and xanthophylls, both of which exhibited similar inhibitory effects on the MAPK and NF-κB signaling pathways in IFN-γ or TNF-α-stimulated HaCaT cells [93]. These findings suggest that astaxanthin and xanthophylls may contribute to the anti-inflammatory properties of *P. yezoensis* extract and play an important role in suppressing inflammatory chemokine production in HaCaT cells.

Table 2 presents a concise synthesis of the primary findings covered in this section, offering a clear overview of the studies discussed (Table 2).

### 2.3. Chlorophyta (Green Algae)

#### 2.3.1. *Seaweed fulvescens* (*Capsosiphon fulvescens*)

*Seaweed fulvescens* (*S. fulvescens*), also known as Maesaengi, is a filamentous chlorophyll alga with a distinct flavor [101,102], found in the North Atlantic and Northern Pacific regions, including South Korea and Japan [103]. Previous research has shown that *S. fulvescens* extracts lower cholesterol levels in rats with hypercholesterolemia [104], inhibit melanogenesis [105], and trigger apoptosis in gastric cancer cells [106], while also exhibiting antioxidant properties and other physiological activities [107]. Chlorophyll, the major colored compound, in *S. fulvescens* exhibits anti-inflammatory [108], antiwrinkle [109], anticancer, wound healing, and tissue engineering properties [110].

In one study, the therapeutic effects of *S. fulvescens* extract on AD were evaluated using a mouse model triggered by dermatophagoides farina bodies (DFB) and TNF-α and IFN-γ-activated HaCaT keratinocytes [101]. Compared to the control group, administration of *S. fulvescens* extract at a dosage of 200 mg per mouse significantly suppressed AD symptoms, resulting in enhanced skin conditions, decreased infiltration of inflammatory cells, reduced lymph node size, and diminished levels of proinflammatory cytokines, specifically IL-6 and TNF-α, along with a reduction in their mRNA expression levels [101]. STAT1, a nuclear protein crucial for mediating cytokine actions, is key in treating inflammatory skin diseases [111]. In NC/Nga mice with AD-like dermatitis, *S. fulvescens* administration suppressed STAT1 phosphorylation in dorsal skin lesions compared to the DFB-treated group, indicating reduced inflammation [101]. *S. fulvescens*, at concentrations of 10, 25, and 50 μg/mL, demonstrated a dose-dependent reduction in STAT1 phosphorylation, which subsequently decreased proinflammatory cytokine production in both in vivo and in vitro models, highlighting its role in modulating the IFN-γ/cytokine signaling pathway [101].

In conclusion, *S. fulvescens* extract’s ability to reduce inflammation and suppress cytokine production through the inhibition of STAT1 phosphorylation in AD models makes it a promising natural alternative for AD treatment.

#### 2.3.2. *Chlorella*

*Chlorella* is a type of single-celled green algae, that boasts a nutrient-rich profile containing amino acids, protein, vitamins, dietary fiber, antioxidants, and chlorophylls [112,113]. Its significant protein content and nutritional value have led to its recognition as a potential dietary staple. In East Asian nations like Japan, Taiwan, and Korea, *Chlorella* is used in various culinary creations, including rice dishes, teas, and pancakes [112,114]. Extensive research has uncovered its multifaceted benefits, demonstrating its potential in combating tumors and obesity, and regulating blood sugar levels in diabetic mice [112,115,116,117].

*Chlorella vulgaris* (*C. vulgaris*), a species known for its high protein content (51–58% of dry matter) and various biological functions, has garnered attention for its potential disease-preventive properties, including protective effects against acute hepatic damage and antioxidant activities [118,119]. Additionally, *C. vulgaris* supplementation has been linked to immune-modulating effects, such as reducing IgE production and histamine secretion, while also enhancing the Th1 cell-related immune response [120,121].

In one study, the effects of *C. vulgaris* supplementation on symptoms resembling AD were investigated in NC/Nga mice [112]. Over six weeks, mice were orally administered *C. vulgaris* (in the standard, 3% and 5% *C. vulgaris*-diet) while AD-like symptoms were induced using the topical application of DFB [112]. Results showed significant improvements in mice that were fed a *C. vulgaris* diet, including lower dermatitis scores, reduced epidermal thickness, and increased skin hydration. Histological analysis revealed decreased infiltration of eosinophils and mast cells in the skin of these mice [112]. Furthermore, serum analysis indicated the downregulation of TARC and MDC levels, with reduced mRNA expression of IL-4 and IFN-γ following *C. vulgaris* supplementation [112].

Recent research into chlorophyll a (Chl a) and its optimized derivative, compound 4aa, reveals promising therapeutic potential for AD via the modulation of Th2 cell pathways [122]. In NC/Nga mouse models of AD, oral administration of Chl a significantly reduced AD-like symptoms, including inflammation and elevated biomarkers (IL-4, IL-17, and IgE), demonstrating effects comparable to those of levocetirizine [122]. Preliminary human clinical trials further supported these findings, showing reductions in clinical AD severity, measured by SCORAD, along with decreased Th2 cell prevalence, suggesting Chl a’s potential in modulating AD-related immune responses [122].

Through metabolic profiling, pheophorbide a (Pa) was identified as an active Chl a metabolite with targeted effects on Th2 differentiation [122]. Structural optimization of Pa led to the development of compound 4aa, which showed enhanced in vitro inhibition of Th2 differentiation, achieving an IC50 of 1.3 μM [122]. This tailored derivation of Pa highlights a novel strategy for refining immune modulation in AD. Mechanistically, 4aa inhibits IL-4 receptor (IL-4R) dimerization, a key step in Th2 differentiation, thereby reducing the expression of GATA3 (a principal Th2 transcription factor) and IL-4 [122]. In vitro, this inhibition demonstrated a potent IC50 of 0.48 μM. As an IL-4R-modulating small molecule, 4aa represents a first-of-its-kind therapeutic approach for AD management, emphasizing specificity in targeting immune pathways [122]. In the DNCB-induced AD mouse model, oral administration of 4aa (5 mg/kg/day) notably alleviated visible symptoms and behaviors related to AD, such as scratching, while significantly lowering levels of key biomarkers (IL-4, IgE, and TSLP) [122]. The therapeutic effects paralleled those of dexamethasone, with additional findings indicating a decrease in IL-4+ cell populations in lymphoid organs, suggesting a reduction in the Th2-dominated immune response commonly seen in AD [122]. In conclusion, compound 4aa emerges as a promising orally available small molecule that could offer a targeted and safe alternative to conventional AD treatments, particularly valuable for patients in need of long-term management solutions. Further clinical studies are essential to assess 4aa’s therapeutic viability and to address unmet needs in AD care. Collectively, these findings underscore the potential of Chl a derivatives and extracts as robust candidates for managing AD symptoms through their immunomodulatory and anti-inflammatory effects.

#### 2.3.3. *Chaetomorpha linum*

*Chaetomorpha linum* (*Müller*) *Kützing* (*C. linum*), originally known as *Conferva linum O.F. Müll.*, is a species of macroalgae belonging to the *Chlorophyceae* family [123,124]. This green seaweed, characterized by its unbranched filaments, is part of the widely distributed *Chaetomorpha* genus [123,124]. Due to its diverse range of bioactive compounds, *C. linum.* is a promising candidate for studying antioxidant properties [123,124].

One study identified various bioactive metabolites in *C. linum.*, including lipids, amino acids, and other compounds with potential anti-inflammatory effects [124]. Phytochemical analysis revealed that *C. linum.* extract inhibited the production of reactive oxygen species (ROS), NO, and prostaglandin E2 (PGE2) in LPS-stimulated RAW 264.7 cells [124]. This inhibition was achieved by reducing inducible nitric oxide synthase (iNOS) and cyclooxygenase-2 (COX-2) expression through the prevention of NF-κB nuclear translocation [124]. Additionally, in TNF-α/IFN-γ-stimulated HaCaT cells, the extract decreased the production of chemokines such as TARC/CCL17, RANTES/CCL5, monocyte chemoattractant protein (MCP)-1/CCL2, and IL-8, as well as the cytokine IL-1β [124]. Based on these findings, *C. linum.* demonstrates strong anti-inflammatory and antioxidant effects, primarily through the inhibition of key inflammatory mediators and signaling pathways like NF-κB. These properties suggest that *C. linum.* holds significant potential as a therapeutic candidate for managing AD, as it targets multiple pathways involved in the inflammatory response associated with AD.

These studies are well-summarized in Table 3, providing a concise overview of key findings discussed in this section.

## 3. Conclusions and Future Prospects

The research discussed in this review underscores the promising therapeutic potential of brown, red, and green algae in addressing the symptoms of AD. Each class of algae contains unique bioactive compounds, such as polysaccharides, phlorotannins, polyphenols, chlorophyll, and sterols, which have demonstrated anti-inflammatory, antioxidant, immunomodulatory, and skin barrier-restoring properties. These compounds work through various mechanisms, including the suppression of pro-inflammatory cytokines, the reduction of IgE levels, and the enhancement of skin barrier function, all of which are crucial in managing AD.

Brown algae, such as *Undaria pinnatifida*, *Costaria costata, Laminaria japonica*, and *Ecklonia cava* have shown strong anti-inflammatory effects by modulating immune responses, reducing epidermal thickness, and lowering histamine and IgE levels. Fucoidan, a sulfated polysaccharide prevalent in many brown algae, has been particularly effective in promoting skin barrier repair and alleviating AD symptoms through immune regulation. Similarly, species of Sargassum have demonstrated the potential to reduce chronic skin inflammation by inhibiting inflammatory mediators and enhancing overall skin health.

Red algae, such as *Sarcodia suiae* and *Chondria crassicaulis*, have also been effective in reducing inflammation and allergic responses by suppressing pro-inflammatory cytokines and IgE production. These algae possess bioactive compounds that contribute to improved skin hydration and the mitigation of AD symptoms, making them promising candidates for therapeutic use.

Green algae, including *Chlorella vulgaris*, *Seaweed fulvescens*, and *Chaetomorpha linum*, offer antioxidant, anti-inflammatory, and immune-modulating properties, making them effective for managing AD. In particular, *Chlorella* and its extracts, especially chlorophyll, have been shown to reduce inflammation, enhance skin condition, and regulate immune responses in AD models.

While in vitro and in vivo studies have demonstrated the potential of these seaweed-derived compounds in managing AD, there is a clear need for more human clinical trials. Future research should focus on evaluating the long-term safety and efficacy of these compounds in human populations. Additionally, further studies are needed to develop optimal formulations and dosing strategies to ensure consistency in therapeutic outcomes. A better understanding of the molecular mechanisms by which these compounds modulate the immune system will also be crucial for advancing their therapeutic applications. Clarifying these mechanisms could result in more targeted treatments for AD and similar chronic skin conditions.

In conclusion, seaweed-based therapies provide a natural, sustainable alternative to conventional AD treatments. By targeting multiple inflammatory pathways and promoting skin health, seaweed-derived compounds hold considerable promise for improving patient outcomes while minimizing the side effects typically associated with pharmaceutical treatments. However, it is also crucial to carefully evaluate the potential risks and side effects of utilizing seaweed for therapeutic purposes. For example, *Sargassum muticum*, a brown alga native to the Pacific coast of Japan, has been reported to secrete phenolic compounds that can cause contact dermatitis [125]. This highlights the importance of comprehensive safety evaluations to ensure that the benefits of seaweed extracts outweigh their potential risks.

Ongoing research into seaweed extracts and their bioactive compounds could lead to the development of novel, safe, and effective treatments for AD and other chronic inflammatory skin disorders. To fully realize this potential, continued focus on innovative extraction technologies, combination therapies, and in-depth safety assessments will be essential in advancing seaweed-derived treatments from research to clinical application.

## Figures and Tables

**Table 1:** Summary of Studies on *Phaeophyceae* (Brown algae).

No.	Type of Seaweed	Solvent	Active Compound	Model (Stimuli)	Conc.	Key Findings	Mechanism	Ref.
1	*Undaria* *pinnatifida*	N/A	Fucoidan	NC/Nga mice (DNCB) HaCaT cell (TNF-α/IFN-γ)	3%	↓ Dermatitis severity scores, epidermal thickness ↓ Mast cell infiltration ↓ Serum IgE and level ↓ TARC, MDC, and RANTES mRNA	Modulation of chemokine expression	[26]
2	*Costaria* *costata*	10% ethanol	N/A	TNC/Nga Mice (DNCB)	100–300 mg/kg	↓ T cell and B cell proliferation and mast cell degranulation ↓ Serum IgE level ↓ IL-4, IL-10, IL-2, IFN-γ, TNF-α and IL-6 ↓ TARC, MDC, RANTES ↓ Eotaxin and TARC mRNA level ↓ IL-4, IL-10 mRNA level	Inhibit expression of Th1, Th2 cytokine and chemokine expression	[29]
3	*Laminaria* *japonica*	Water	N/A	NC/Nga mice (DNCB), HaCaT cell, (TNF-α/IFN-γ)	100–300 mg/mL 25–200 µg/mL	↓ TARC, RANTES ↓ Skin inflammation ↓ pruritus, Skin hydration ↓ p38 MAPK phosphorylation	MAPK–STAT1 signaling pathway	[36]
		70% ethanol	N/A	ICR mice (DNCB) HaCaT cell (TNF-α/IFN-γ)	10–100 mg/kg 0.01–1 mg/mL	↓ Skin erythema, thickness ↓ Serum histamine and IgE levels ↓ IL-6 and IL-8 ↓ Nuclear translocation of NF-κB p65	NF-κB pathway	[37]
4	*Ecklonia* *cava*	N/A	Dieckol	HaCaT cell (TNF-α/IFN-γ)	5–10 µM	↓ MDC/CCL22 ↓ Inflammatory response ↓ Phosphorylation and nuclear translocation of STAT1	STAT1 pathway	[38]
		N/A	Eckol		25–100 μg/mL	↓ IL-6 ↓ MDC and TARC ↓ Phosphorylation of JAK1/STAT1/NF-κB	JAK1/STAT1/ NF-κB pathway	[48]
		N/A		HaCaT cell (TNF-α/IFN-γ)	25–100 μg/mL	↓ IL-1β, IL-4, IL-5, IL-6, IL-13, TNF-α and IFN-γ ↓ MDC, TARC, RANTES, and eotaxin ↓ Phosphorylation of MAPK and NF-κB	MAPK and NF-κB signaling pathways	[39]
5	*Cladosiphon okamuranus*	N/A	Fucoidan	BALB/c mice (DNCB) P815 mast cell (C48/80)	200–800 mg/kg/day 75–600 µg/mL	↓ IL-4, IL-5, IL-6, IL-13, IL-22 and TSLP ↓ SCORAD index ↓ Lymph node weight ↓ Mast cell degranulation	Regulation of pro-inflammatory cytokine	[52]
		N/A		BALB/c mice (DNCB) RAW264.7 cell (LPS)	3.5–7% 31.25–1000 μg/mL	↓ IL-4, IL-5, IL-22, IL-33, and TSLP ↓ NO production ↓ Total serum IgE level ↓ Th22 and memory B cells ↑ TGF-β1 ↑ Treg cell differentiation ↑ Skin regeneration and epidermal thickness	Modulation of immune response and cytokine profile	[53]
6	*Fucus* *vesiculosus*	N/A	Fucoidan	BALB/c mice (DNFB)	0.2%	↓ TNF-α and IL-12 ↓ Serum IgE and IL-4 ↓ CD4+ T cell proliferation ↓ Skin erythema, Epidermal thickness and hydration ↑ IL-10 and TGF-β ↑ SR-A mRNA level	CD4+ T cell proliferation inhibition via SR-A interaction	[56]
7	*Sargassum* *horneri*	70% ethanol	N/A	BALB/c mice (TPA) HaCaT cell (TNF-α/IFN-γ)	62.5 μg/mL 15.6–62.5 μg/mL.	↓ TSLP, IL-4, IL-6, IL-13, IFN-γ and TNF-α ↓ Ear edema, epidermal hyperplasia ↓ Inflammatory cell infiltration ↓ mRNA levels of IL-25, IL-33 TSLP, IL-1β, IL-4, IL-6, IL-13, IFN-γ and TNF-α ↓ Eotaxin, MDC, RANTES and TARC mRNA expression	Inhibition of MAPK/NF-κB and Nrf2/HO-1 signaling pathway	[61]
		70% ethanol	N/A	NC/Nga mice (DNCB)	10–100 mg/kg	↓ Epidermal thickness ↓ Mast cell proliferation ↓ Serum IgG1, IgG2a ↓ TSLP and IL-13 mRNA expression ↑ Filaggrin expression	Skin recovery via enhanced filaggrin protein expression	[62]
		Ethanol	N/A	NC/Nga mice (HDM/DNCB)	10–100 mg/kg	↓ TEWL, ear edema ↓ Mast cell and eosinophil infiltration ↓ Serum IgG 1 and IgG 2a levels ↓ IL-4, IL-5, IL-6, IL-10, IL-13, and IFN-γ mRNA expression ↓ TARC mRNA level	Suppression of inflammatory cytokines	[63]
		Water	N/A	BALB/c mice (DNCB) Splenocytes (DNCB)	30 mg/mL 0.1–100 μg/mL	↓ Epidermal thickness ↓ Inflammatory cell infiltration ↓ IL-4 and IL-5 ↓ Serum IgE level ↑ IFN-γ Cytokine	Restoration of the Th1/Th2 balance	[64]
8	*Sargassum fulvellum*	95% ethanol	N/A	BALB/c mice (DNCB) Splenocytes (DNCB)	30 mg/mL 0.1–100 μg/mL	↓ Skin erythema, edema, dryness, epidermal thickness ↓ Inflammatory cell infiltration ↓ Serum IgE, TNF-α, and IL-4 levels ↑ Serum IL-10 level ↓ Splenocyte IL-4, IL-5, and IL-13 levels ↑ Splenocyte IFN-γ level	Regulation of Th1/Th2 cytokines and inhibition of inflammatory cell infiltration	[65]
		N/A	Grasshopper ketone	Splenocytes (ConA)	0.1–100 μg/mL	↓ IL-4 and IFN-γ		
9	*Sargassum muticum*	N/A	Apo-9′-fucoxanthinone	BALB/c mice (DNCB) CD4+ T cell (DNCB)	100 mg/kg 6.25–50 μM,	↓ Ear edema ↓ Inflammatory cell infiltration ↓ Serum IgE level ↓ Lymph node edema ↓ IFN-γ, TNF-α, and IL-4 mRNA levels	Inhibition of pro-inflammatory cytokines	[66]
10	*Sargassum hemiphyllum*	Methanol	N/A	ICR mice (Anti-DNP IgE/DNP-HSA) HMC-1 (PMA/A23187) RPMCs (compound 48/80)	0.1–1.0 g/kg 0.01–1 mg/mL 10–100 μg/mL	↓ Skin anaphylactic reaction ↓ IL-8 and TNF-α ↓ Histamine and β-hexosaminidase release	Suppression of mast cell activation and inflammatory mediator production	[67]
11	*Sargassum* *serratifolium*	Ethanol	SCM SQA SHQA	BALB/c mice (DNCB) HR-1 mice (DNCB) RAW 264.7 (LPS) HaCaT cell (TNF-α)	500–2000 ppm 2.5–5 ppm	↓ Epidermal thickness ↓ Mast cell infiltration ↓ NO production ↓ IL-6, COX-2, and TNF-α mRNA levels	NO production inhibition and inhibition of inflammation-related cytokine mRNA levels	[68]
12	*Hizikia* *(Sargassum) fusiformis*	A butanoic acid-rich fraction	Fucosterol	BALB/c mice (DNCB) NC/Nga mice (DNCB) RAW 264.7 (LPS) mouse plenocytes (anti-CD3 mAb)	1.25–2.5 mg/kg 200 mg/kg weight/day	↓ AD symptoms, cell infiltration and T cell activation ↓ serum IgE ↓ IFN-γ, IL-2, IL-4 and TNF-α in the media of splenocytes ↓ Phosphorylation of NFAT ↓ IL-4 and IL-5 mRNA expression in splenocytes	Inhibition of helper T cell-dependent cytokine expression in splenocytes. Rebalancing of the Th1/Th2 immune response	[69]
13	*Ishige* *okamurae Yendo*	70% ethanol extracts	diphlorethohydroxycarmalol	BALB/c mice (DNCB/HDM) HaCaT cell (TNF-α/IFN-γ)	50–100 mg/kg 3–30 μg/mL	↓ IL-1β, IL-6, IL-8, TNF-α, TARC/CCL17, CCL22/MDC ↓ Serum IgE ↓ Skin lesions, ear edema, immune cell and mast cell infiltration ↓ Serum levels of IgE, IgG1, and IgG2a	MAPKs /STAT-1 /NF-κB pathways	[83]

DNCB, 1-Chloro-2,4-dinitrobenzene; TNF, tumor necrosis factor; IFN, interferons; TARC, thymus activation regulated chemokine; MDC, macrophage-derived chemokine; RANTES, regulated upon activation normal T cell expressed and secreted; IL, interleukin; Th, T helper; MAPK, mitogen-activated protein kinase; STAT, signal transducer and activator of transcription; NF-κB, nuclear factor-kappa B; CCL, C-C motif chemokine ligand; JAK, janus kinase; SCORAD, scoring atopic dermatitis; HDM, house dust mite; Ig, immunoglobulin; TEWL, transepidermal water loss; TSLP, thymic stromal lymphopoietin; DNP, dinitrophenyl; HAS, Human Serum Albumin; phorbol 12-myristate 13-acetate, PMA; COX-2, cyclooxygenase-2; NO, nitric oxide; regulatory T cells; TGF, transforming growth factor; TPA, O-tetradecanoylphorbol-13-acetate; HMC, human mast cell line; RPMCs, rat peritoneal mesothelial cells; LPS, lipopolysaccharide; SCM, sargachromanol; SQA, sargaquinoic acid; SHQA, sargahydroquinoic acid; NFAT, nuclear factor of activated T cell; N/A, not applicable; ↑, increase; ↓, decrease.

**Table 2:** Summary of Studies on *Rhodophyta* (Red algae).

No.	Type of Seaweed	Solvent	Active Compound	Model (Stimuli)	Conc.	Effect	Mechanism	Ref.
1	*Sarcodia* *suiae* sp.	Ethyl acetate fraction	N/A	BABL/c mice (DNCB)	50 µg/200 µg in 100 µL methylcellulose	↓ Epidermal thickness ↓ Serum IgE ↓ Spleen and lymph node edema ↓ Mast cell infiltration	Increased expression of claudin-1 and filaggrin	[90]
2	*Chondria* *crassicaulis*	95% ethanol	N/A	BABL/c mice (DNCB) Splenocytes (DNCB) RBL-2H3 cell (DNP-BSA)	30 mg/mL	↓ Severity of dermatitis ↓ skin scarring, edema, and scratching ↓ Serum IgE ↓ Mast cell degranulation, ↓ IL-4 and IL-5 ↑ IFN-γ	Decreased levels of Th2 cytokines and increased levels of Th1 cytokines	[92]
3	*Porphyra* *yezoensis*	80% methanol	Astaxanthin/ Xanthophyll	HaCaT cell (IFN-γ/TNF-α)	40–1000 mg/mL	↓ TARC and MDC mRNA expression ↓ p-ERK, p-JNK, and p-p38 protein levels	Inhibition of MAPK and NF-κB signaling pathways	[93]

DNCB, 1-Chloro-2,4-dinitrobenzene; Ig, immunoglobulin; DNP, dinitrophenyl; BSA, bovine serum albumin; IL, interleukin; TNF, tumor necrosis factor; IFN, interferons; Th, T helper; TARC, thymus activation regulated chemokine; MDC, macrophage-derived chemokine; ERK, extracellular signal-regulated kinases; JNK, c-Jun N-terminal kinases; MAPK, mitogen-activated protein kinase; NF-κB, nuclear factor-kappa B; N/A, not applicable; ↑, increase; ↓, decrease.

**Table 3:** Summary of Studies on *Chlorophyta* (green algae).

No.	Type of Seaweed	Solvent	Active Compound	Model (Stimuli)	Conc.	Key Findings	Mechanism	Ref.
1	*Seaweed fulvescens*	70% ethanol extracts	N/A	NC/Nga mice (DFB) HaCaT cell (TNF-α/IFN-γ)	200 mg/mouse 10–50 µg/mL	↓ Mercmal score and skin lesion symptoms ↓ TNF- α, IL-6 production and mRNA expression levels ↓ STAT1 phosphorylation ↓ Cellular infiltration and lymph node size	Cytokine reduction through STAT1 pathway	[101]
2	*Chlorella*	N/A	N/A	NC/Nga mice (DFB)	3–5%	↓ Dermatitis score and skin lesion symptoms ↓ Serum TARC, MDC ↓ IL-4 and IFN-γ mRNA level	Modulating via cytokine expression level	[112]
		N/A	Chl a 4aa	NC/Nga mice (DNCB)	5 mg/kg/day	↓ Skin lesion symptoms ↓ IL-4, IgE and TSLP levels	Downregulation Gata3 and IL-4 expression levels in Th2 cells by blocking IL-4 receptor dimerization.	[122]
				Human patients with AD	25 mg/day	↓ SCORAD value ↓ CD4+ IL-4+ Th2 cells		
3	*Chaetomorpha linum*	70% Ethanol	N/A	RAW 264.7 (LPS) HaCaT cell (TNF-α/IFN-γ)	25–100 µg/mL	↓ ROS, NO, and PGE2 production ↓ iNOS/COX-2 expression ↓ NF-κB nuclear translocation ↓ TARC/CCL17, RANTES/CCL5, MCP-1/CCL2, IL-8 and IL-1β	Downregulation through NF-κB pathway	[124]

DFB, dermatophagoides farina bodies; DNCB, 1-Chloro-2,4-dinitrobenzene; TNF, tumor necrosis factor; IFN, interferons; TARC, thymus activation regulated chemokine; MDC, macrophage-derived chemokine; RANTES, regulated upon activation normal T cell expressed and secreted; IL, Interleukin; STAT, signal transducer and activator of transcription; NF-κB, nuclear factor-kappa B; CCL, C-C motif chemokine ligand; Ig, immunoglobulin; TSLP, thymic stromal lymphopoietin; SCORAD, scoring atopic dermatitis; NO, nitric oxide; LPS, lipopolysaccharide; ROS, reactive oxygen species; iNOS, inducible nitric oxide synthase; COX, cyclooxygenase; PGE2, prostaglandin E2; MCP, monocyte chemoattractant protein; N/A, not applicable; ↑, increase; ↓, decrease.

## Data Availability

Data sharing does not apply to this article as no new data were created or analyzed in this study.
